# Caecal appendix lipomatosis in a pregnant patient mimicking acute appendicitis

**DOI:** 10.31744/einstein_journal/2020RC5415

**Published:** 2020-11-25

**Authors:** Laura Pereira Sanches, Antônio Rahal, Priscila Mina Falsarella, Vithor de Oliveira Carvalho, Leonardo Guedes Moreira Valle, Miguel José Francisco, Rodrigo Gobbo Garcia, Marcelo Buarque de Gusmão Funari

**Affiliations:** 1 Hospital Israelita Albert Einstein São PauloSP Brazil Hospital Israelita Albert Einstein, São Paulo, SP, Brazil.

**Keywords:** Ultrasonography, Lipomatosis, Appendicitis, Magnetic resonance imaging, Diagnosis, differential, Pregnant women

## Abstract

A 34-years-old pregnant woman admitted in the emergency unit complaining about worsening right iliac fossa pain for 2 days. Acute appendicitis was the suspected diagnosis. Laboratory exams were ordered and results were within normal limits for infectious and inflammatory aspects. Ultrasound scan revealed a pregnancy in course without alterations and a thickness of the appendix wall without inflammatory signs in the surrounding tissue. Because the suspicion of acute appendicitis remained, a magnetic resonance was done and confirmed the diagnosis of a cecal appendix lipomatosis.

## INTRODUCTION

Lipomatosis is the most frequent benign condition resulting from abnormal and increased proliferation of mature fat tissue in submucosal layers in the intracellular compartment. The more frequent clinic presentation of this condition is central medium portion of the body with predominance in the cephalic pole, more specifically the head, as well as in the dorsum. This problem is often related to the nodule or infiltrated unpainful tissue, which can cause symptoms secondary to the regional compression of joints or due to the rapid growth.^(^^1^^)^

Lipomatosis with gastrointestinal presentation is, among all forms, the less frequent condition, and may occur in any portion of digestive tract, and the most frequent locations of these injuries of the colon, ileum, and jejunum.^(^^2^^)^ In addition, lipomatosis may be presented in diffuse, asymmetric, or focal forms. When diffuse presentation may course with intestinal occlusion in different intensities, including total occlusion, digestive hemorrhage, diarrhea, vomiting, nausea, constipation, and pain in a variety degrees.^(^^3^^)^ In local or localized form, only few patients present symptoms and, in general, in asymptomatic cases, the lipoma exceeds 2cm in diameter.^(^^4^^,^^5^^)^

The goal of this report was to describe a rare manifestation of focal intestinal lipomatosis in the caecal appendix in a pregnant woman with low abdominal pain, as well as to provide a detailed description of main characteristics of this disease both in static and dynamic B-mode and doppler velocimetry ultrasound and sectional methods, such as computed tomography (CT) and magnetic resonance imaging (MRI). We believe that, considering the multiplicity of the presentation and the rarity of this specific form, to list characteristics of imaging in different methods may contribute to the practice of radiologists, particularly to those professionals working on emergency radiology by avoiding incorrect diagnosis, as well as unnecessary clinical-surgical management.

## CASE REPORT

A 34-year-old women in the second trimester of pregnancy was admitted to the emergency unit complaining about intensive moderate pain on lower portion of the abdomen with predominance to the right side for 3 weeks that worsened in the last 2 days. The patient did not present other symptoms such as fever, inappetence, nausea, or vomiting. Based on the abdominal pain diagnosis with predominance in the right Iliac fossa, laboratorial exams and a total abdomen ultrasonography (USG) were requested in the radiology emergency unit to assess inflammatory and infectious disease activities.

**Figure 1:**
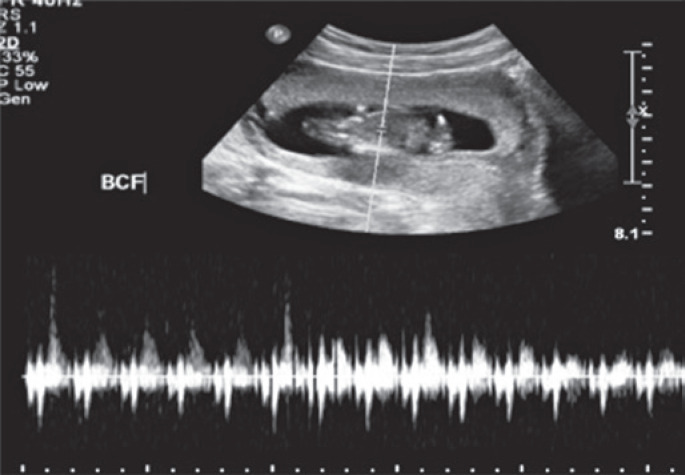
Early pregnancy. Topic fetus with normal heart rate

**Figure 2:**
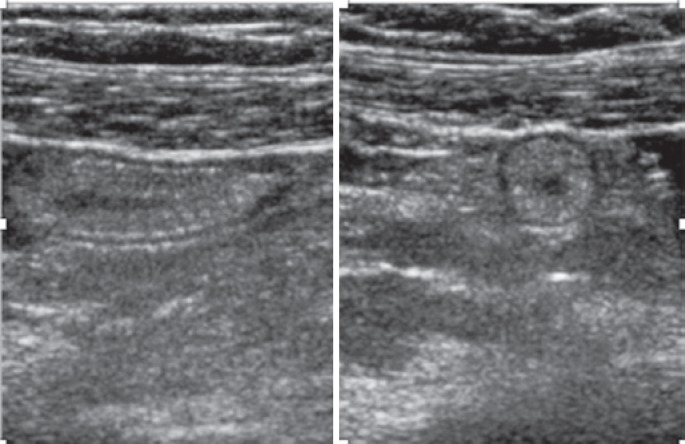
Caecal appendix with thicker wall identified in the ultrasonography

The USG confirmed the pregnancy without evident alterations related to the pregnant uterus, to the placenta, and to the fetus ( Figure 1 ). The directed evaluation of the right iliac fossa did not characterize suggestive signs of acute appendicitis. In this context, these signs would be hyperechogenicity and densification of the pericecal fat, locoregional lymphonodomegaly of reactional aspect with cortical thickness and, perhaps, the increase of the flow mapping with colour Doppler, and the presence of laminar free liquid, or locoregional collection. Caecal appendix was thicker in the body portion, reaching 9.2mm of maximal transverse diameter (the normal value is considered up to 6.0mm) and segment increase of the caliber in this region, without other inflammation characteristics ( Figure 2 ). In the thickened region, the light of the appendix was collapsed, and the wall of the organ had an increased echogenicity, in a homogeneous format, and with similar echotexture to fat. The appendicular compressibility was slightly reduced. In the appendicular apex, the external diameter was within normal limits (4.5mm). In the Doppler colour mapping no increase of flow was observed. In addition, no signs of hypercogenicity of periappendicular fat were seen.

The laboratorial exams did not show alterations (leukogram and C-reactive protein values were within normal ranges). Findings from the USG was not suggestive to acute inflammatory process, however they showed changes with benign aspects probably due to the fatty infiltration. A MRI exam was requested to complement the diagnosis.

**Figure 3:**
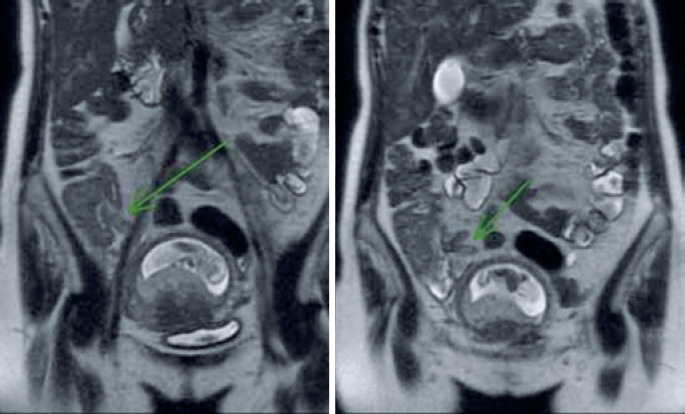
Coronal plane magnetic resonance imaging. The arrows point to cecal appendix with thickened wall

**Figure 4:**
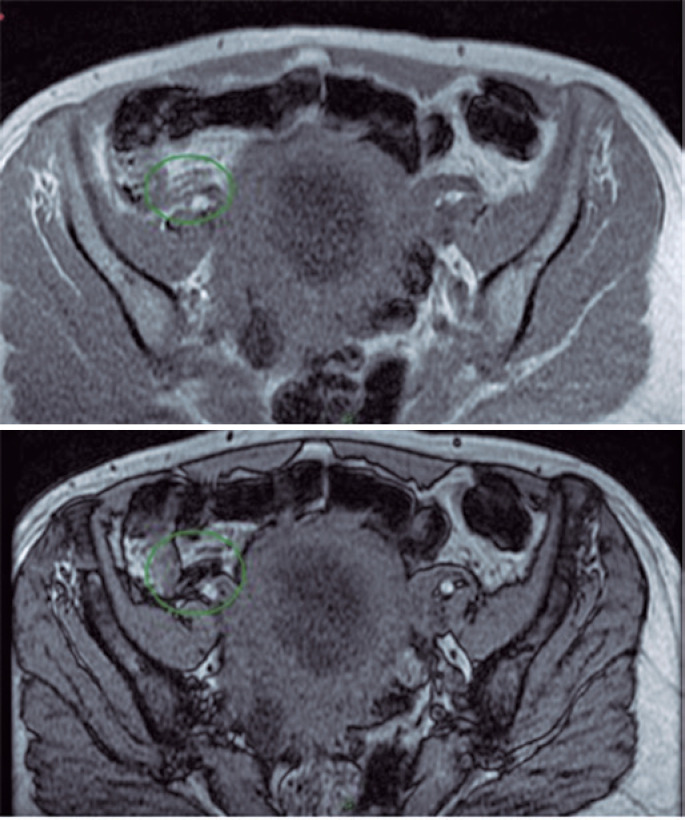
Cecal appendix with thicker wall showed in the axial plan magnetic resonance imaging

**Figure 5:**
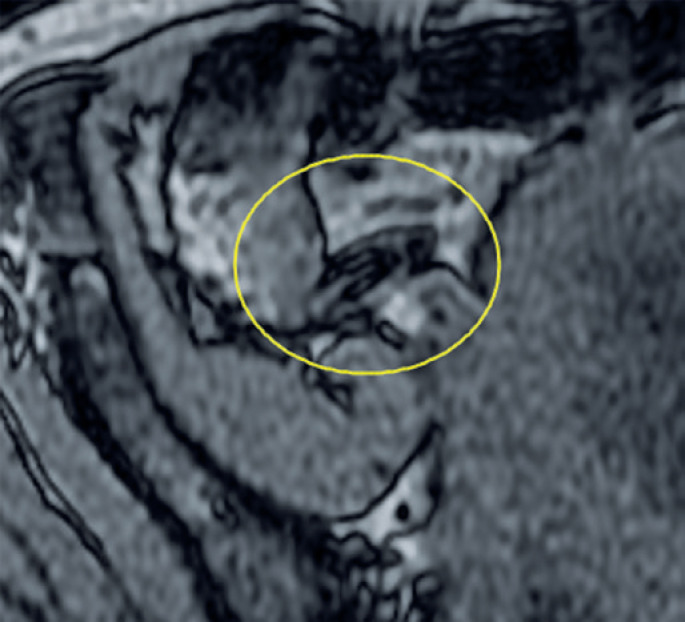
Cecal appendix showing drop on out-of-phase sequence sign, and showing fatty component

The MRI confirmed the findings described in the USG with focal appendicular parietal thickness without inflammatory signs ( Figures 3 and 4 ). We observed diffuse hyper signal of appendix on in-phase sequence with loss of signal on out-phase sequence ( Figure 5 ), therefore, indicating the presence of expressive amount of fat in intracellular compartment; a characteristic of lipomatosis. No significant highlight in the appendix was observed from the MRI exam. The clinical and laboratory findings, the USG, and MRI enabled to confirm the diagnosis of lipomatosis. The patient was discharged with analgesia, and return of symptoms was not encountered during follow-up.

## DISCUSSION

Among differential diagnosis of right iliac fossa pain are included a number of conditions such as appendicitis, diverticulitis, distal ureteral lithiasis, Crohn's disease, ovarian-tube inflammatory diseases, and ectopic pregnancy.^(^^6^^)^ In this report, the MRI was conducted exclusively with the aim to confirm the non-inflammatory and benign nature of the appendicular injury in one pregnant patient.

The first report on intestinal lipomatosis focusing on caecal appendix dates back from 1956 in a study by Antoci, and in his study the definite final diagnosis was conducted by histopathologic analysis.^(^^7^^)^ Among appendicectomies, 4.5% to 15% are negative for appendicitis (with higher incidences of surgeries without inflammation findings in women).^(^^8^^)^ Such consideration is still relevant when findings from images are not conclusive for acute appendicitis and/or when inflammatory proofs are normal.^(^^8^^)^

Among currently available medical imaging methods, the USG is of the most commonly adopted exam in the emergency unit due to its low cost exam, little invasiveness, no radiation - a relevant aspect for women and, particularly, pregnant women - and with good diagnostic accuracy in experienced hands.^(^^9^^)^ One of the main limitations is the fact that this exam is operating dependent, which makes the knowledge by radiologists of imaging aspects of this condition even more important.^(^^9^^)^

The CT also presents good accuracy, however, the use of ionizing radiation limits its application among pregnant women (as in the present case report) or among women wishing to become pregnant. The CT constitutes the most available, less expensive, and fastest method compared with the MRI.^(^^10^^)^ The MRI is indicated, mainly, for patients with inconclusive ultrasonography findings and for those that ionizing radiation should be avoided.^(^^10^^)^

Findings of intestinal lipomatosis image vary based on the imaging method chosen, and the radiologist must recognize the different aspects in each one of the techniques. In the USG, intestinal lipomas presents as characterized marks the regular and homogenous parietal thickness with hyperechogenicity that derivates from presence of fat in submucosa layer. The decrease of compressibility of the organ with the use of transductor is present, however, this is quite less relevant compared with one inflamed appendix – a true acute appendicitis.

In the CT, the characteristic of the image is a hypodense homogenous mass with attenuation between -80 to -120 units of the Hounsfield.^(^^3^^)^ In the case of intestinal lipomatosis, this aspects check in the within the appendiceal wall that is due to the fat infiltration that present hypoattenuating in a homogenous mode.

In the MRI, the great specificity that determines the diagnosis of lipomatosis is the high intensity of the sign in images weighted in T1 and T2, with decrease in intensity of the signal in sequence with fat suppression. We obtained the definition of the presence of intracellular fat in intestinal wall, especially on in/out-phase series, which led to definition of the diagnosis without the need of the histological sample. The lack of malignity, *i.e* ., a malign inheritance of fat suppression, such as in case of liposarcoma instead of lipomatosis, occur for regularity and parietal homogeneity, presence of well-defined clival plans with fat plans, presence of adjacent organs and, particularly, lack of highlighting in the sequence after the use of gadolinium.^(^^11^^)^

## CONCLUSÃO

Intestinal lipomatosis with focus in caecal appendix is an uncommon and rare condition associated with locoregional symptoms. Given the rarity of this disease, in some cases the diagnosis may not be conducted particularly due to the mild clinical findings and the often incidental diagnosis. Radiologists have a central role in the diagnosis workup, for this reason, they must understand aspects of images in a variety of methodologies, particularly ultrasonography, computed tomography, and magnetic resonance imaging. Given the prevalence of acute appendicitis in the emergency care, to differentiate an acute inflammatory process requiring intervention from a benign condition with a traditional follow-up is of highly importance both for an assertive report as well as to avoid invasive and unnecessary treatments.
