# Supplementary material for: Caecal appendix lipomatosis in a pregnant patient mimicking acute appendicitis
Source: Einstein (Sao Paulo). 2020 Nov 25;18:eRC5415. doi: 10.31744/einstein_journal/2020RC5415 (PMC7690936; doi:10.31744/einstein_journal/2020RC5415)
Supplement: Translation

# Translation: Lipomatose do apêndice cecal em paciente grávida mimetizando apendicite aguda

**Authors:** Laura Pereira Sanches, Antônio Rahal, Priscila Mina Falsarella, Vithor de Oliveira Carvalho, Leonardo Guedes Moreira Valle, Miguel José Francisco, Rodrigo Gobbo Garcia, Marcelo Buarque de Gusmão Funari

**Affiliations:** 1 São PauloSP Brasil Hospital Israelita Albert Einstein, São Paulo, SP, Brasil.

**Keywords:** Ultrassonografia, Lipomatose, Apendicite, Imagem por ressonância magnética, Diagnóstico diferencial, Gestantes

## Abstract

Paciente de 34 anos, do sexo feminino, grávida, chega ao pronto-socorro com queixa de dor na fossa ilíaca direita piorando nos últimos 2 dias com suspeita de apendicite aguda. Foram solicitados exames laboratoriais, que estavam dentro dos limites de normalidade para aspectos infecciosos e inflamatórios. Exame de imagem também foi solicitado, sendo a ultrassonografia o método de escolha, que revelou gravidez em curso sem alterações e espessura da parede do apêndice sem sinais inflamatórios. Ainda com suspeita de apendicite aguda, foi realizada ressonância magnética, confirmando a hipótese de lipomatose do apêndice cecal.

## INTRODUÇÃO

A lipomatose é uma condição mais frequentemente benigna, resultante da proliferação anormal e pronunciada de tecido adiposo maduro em camadas submucosas, no compartimento intracelular. Sua apresentação clínica mais frequente é na porção central mediana do corpo, com predomínio no polo cefálico, mais especificamente na cabeça, bem como no dorso.

Trata-se, geralmente, de nódulo ou infiltrado tecidual indolor, podendo causar sintomas secundários à compressão regional de articulações ou decorrente de crescimento rápido.^(^^1^^)^

A lipomatose com apresentação gastrintestinal é, dentre todas as formas, a condição menos frequente, podendo ocorrer em qualquer porção do trato digestivo, sendo as localizações mais frequentes dessas lesões o cólon, o íleo e o jejuno.^(^^2^^)^

A lipomatose intestinal pode se apresentar nas formas difusa, salteada ou focal. Quando na apresentação difusa, pode cursar com oclusão intestinal, em diferentes intensidades, inclusive com quadros totalmente oclusivos, hemorragia digestiva, diarreia, vômitos, náuseas, constipação e dor, em graus variados.^(^^3^^)^Na forma focal ou localizada, apenas uma minoria dos pacientes apresenta sintomas e, em geral, nos casos sintomáticos, os lipomas excedem 2cm em diâmetro.^(^^4^^,^^5^^)^

O objetivo deste relato foi descrever uma manifestação bastante rara da lipomatose intestinal focal no apêndice cecal em paciente do sexo feminino, gestante, com quadro de dor abdominal no baixo ventre, bem como pormenorizar as principais características de imagem dessa patologia, tanto ultrassonográficas, no modo B estático e dinâmico e dopplervelocimetria, quanto em métodos seccionais, como tomografia computadorizada (TC) e ressonância magnética (RM). Acreditamos que, considerando a multiplicidade de apresentações e a raridade dessa forma específica, listar as características de imagem nos diferentes métodos pode contribuir sobremaneira com radiologistas de modo geral, sobretudo aqueles com foco em radiologia de emergência, evitando diagnósticos equivocados, bem como condutas clínico-cirúrgicas desnecessárias.

## RELATO DE CASO

Paciente do sexo feminino, com 34 anos, no segundo trimestre de gestação, chega ao pronto atendimento com queixa de dor de moderada intensidade no andar inferior do abdome, com predomínio à direita, há 3 semanas, com piora acentuada nos últimos 2 dias. A paciente não apresentava outros sintomas além da dor referida, como febre, inapetência, náuseas ou vômitos. Diante do diagnóstico de dor abdominal com predomínio na fossa ilíaca direita, foram solicitados exames laboratoriais, para avaliação de atividade inflamatória e infecciosa, e ultrassonografia (USG) de abdome total, no setor de radiologia de emergência.

A USG confirmou o quadro de gestação em curso, sem alterações evidentes relacionadas ao útero gravídico, à placenta e ao feto ( Figura 1 ). A avaliação direcionada para a fossa ilíaca direita não caracterizou sinais sugestivos de apendicite aguda que, nesse contexto, seriam hiperecogenicidade e densificação da gordura pericecal e/ou periapendicular, linfonodomegalia locorregional de aspecto reacional com espessamento cortical e, por vezes, aumento de fluxo ao mapeamento com Doppler colorido e presença de líquido livre laminar ou sob forma de coleção locorregional. O apêndice cecal encontrava-se espessado na porção corporal, atingindo 9,2mm de diâmetro transverso máximo (valor considerado normal até cerca de 6,0mm) e com aumento segmentar do calibre nessa região, sem outras características de natureza inflamatórias ( Figura 2 ). Na região espessada, a luz do apêndice estava colabada, e a parede do órgão apresentava ecogenicidade aumentada, de modo homogêneo, e com ecotextura semelhante à da gordura. A compressibilidade apendicular estava levemente reduzida. Na região do ápice apendicular, o diâmetro externo apresentava-se dentro dos limites de normalidade (4,5mm). Ao mapeamento com Doppler colorido, não foi observado aumento de fluxo. Também não existiam sinais de hiperecogenicidade da gordura periapendicular.

**Figura 1:** Gravidez precoce em curso. Feto tópico com batimentos cardíacos fetais regulares

**Figura 2:** Apêndice cecal com parede espessada identificada na ultrassonografia

Os exames laboratoriais não evidenciaram alterações (leucograma e proteína C-reativa com valores dentro da faixa de normalidade). O conjunto de achados evidenciados à USG não foi sugestivo de processo inflamatório agudo, mas de alterações de aspecto benigno de provável natureza infiltrativa gordurosa. Foi solicitado exame de RM para complementar o diagnóstico.

A RM confirmou os achados descritos na USG, com espessamento parietal apendicular focal sem sinais inflamatórios ( Figuras 3 e 4 ). Foi observado hipersinal difuso do apêndice na sequência *in-phase* , com perda de sinal na sequência *out-phase* ( Figura 5 ), indicando a presença de quantidade expressiva de gordura no compartimento intracelular, característica de lipomatose. Não houve realce apreciável no apêndice no exame de RM. Face ao conjunto dos achados clínico-laboratoriais e às características da USG e da RM, o diagnóstico de lipomatose pôde ser confirmado. A paciente foi liberada com analgesia, sem retorno dos sintomas.

**Figura 3:** Ressonância magnética no plano coronal. A seta aponta para o apêndice cecal, com sua parede espessa

**Figura 4:** Apêndice cecal com parede espessa visualizada no plano axial na ressonância magnética

**Figura 5:** Apêndice cecal evidenciando queda do sinal na sequência *out-phase* , demonstrando componente gorduroso

## DISCUSSÃO

Dentro dos diferenciais de dor na fossa ilíaca direita, são incluídas diversas condições, como apendicite, diverticulites, litíase ureteral distal, doença de Crohn, doenças inflamatórias tubo-ovarianas e gravidez ectópica.^(^^6^^)^A RM fora realizada, neste contexto, exclusivamente com a finalidade de confirmar a natureza não inflamatória e benigna da lesão apendicular em uma paciente gestante.

O primeiro relato da lipomatose intestinal com foco no apêndice cecal data de 1956 por Antoci, sendo o diagnóstico final definido por meio de análise histopatológica.^(^^7^^)^Dentre as apendicectomias, 4,5% a 15% são negativas para apendicite (com maior incidência das cirurgias sem achados de inflamação no sexo feminino).^(^^8^^)^Tal consideração é ainda mais relevante quando os achados de imagem não são conclusivos para apendicite aguda e/ou as provas inflamatórias são normais.^(^^8^^)^

Dentro do rol de métodos de imagem disponíveis atualmente, a USG desponta como exame cada vez mais utilizado na emergência, visto que apresenta baixo custo, é pouco invasiva e sem radiação – aspecto particularmente relevante em pacientes do sexo feminino e grávidas –, com boa acurácia diagnóstica, em mãos experientes.^(^^9^^)^Uma de suas principais limitações é o fato de ser um exame operador-dependente, o que torna ainda mais importante o conhecimento dos aspectos de imagem dessa condição pelos médicos radiologistas.^(^^9^^)^

A TC também apresenta boa acurácia diagnóstica, mas o uso de radiação ionizante limita sua realização em pacientes gestantes (como no caso em questão) ou com possibilidade de gravidez. Trata-se do método mais disponível, menos oneroso e de mais rápida realização que a RM.^(^^10^^)^A RM é reservada, mais frequentemente, aos pacientes com achados ultrassonográficos inconclusivos e nos quais a radiação ionizante deve ser evitada.^(^^10^^)^

Os achados de imagem na lipomatose intestinal variam de acordo com o método de imagem empregado, devendo o radiologista conhecer os diferentes aspectos em cada uma das técnicas. Na USG, os lipomas intestinais apresentam, como marcas características, espessamento parietal homogêneo e regular, com hiperecogenicidade, que deriva da presença de gordura no plano da camada submucosa. A redução da compressibilidade do órgão com o uso do transdutor está presente, todavia bastante menos relevante quando em comparação com um apêndice inflamado – uma verdadeira apendicite aguda.

Na TC, a característica de imagem é a de massa homogênea hipodensa com atenuação entre -80 a -120 unidades de Hounsfield.^(^^3^^)^No caso de lipomatose intestinal, esse aspecto é verificado no interior da parede apendicular, que, devido à infiltração adiposa, apresenta-se hipoatenuante, de modo homogêneo.

Na RM, a grande peculiaridade que determina o diagnóstico de lipomatose é a alta intensidade de sinal nas imagens ponderadas em T1 e T2, com queda da intensidade de sinal nas sequências com supressão de gordura. Especialmente nas series *in/out-phase* , obtemos a definição da presença de gordura intracelular na parede intestinal, fechando o diagnóstico, sem a necessidade de amostragem histológica. A ausência de malignidade, ou seja, de que se trata de uma linhagem maligna de acúmulo de gordura, como, por exemplo, no caso de um lipossarcoma ao invés de lipomatose, dá-se por regularidade e homogeneidade parietais, presença de planos clivais bem definidos com gordura e órgãos adjacentes e, sobretudo, ausência de realce nas sequências após o uso do gadolíneo.^(^^11^^)^

## CONCLUSÃO

A lipomatose intestinal com foco no apêndice cecal é condição pouco frequente e raramente associada a sintomas locorregionais. Dada a raridade dessa apresentação, seu diagnóstico pode, por vezes, não ser realizado, sobretudo devido ao quadro frustro, por vezes com diagnóstico meramente incidental. O radiologista é um profissional fundamental para definir o diagnóstico, mas, para tanto, necessita dominar os aspectos de imagem nas diferentes metodologias, destacando-se a ultrassonografia, a tomografia computadorizada e a ressonância magnética. Dada a prevalência de apendicites agudas no âmbito do pronto-atendimento, saber diferenciar um processo inflamatório agudo, com necessidade de intervenção, de uma condição de curso benigno e com seguimento conservador, é de suma importância, tanto para um relatório assertivo, quanto para evitar condutas invasivas e desnecessárias.
